# Tsbrowse: an interactive browser for ancestral recombination graphs

**DOI:** 10.1093/bioinformatics/btaf393

**Published:** 2025-07-12

**Authors:** Savita Karthikeyan, Ben Jeffery, Duncan Mbuli-Robertson, Jerome Kelleher

**Affiliations:** Big Data Institute, Li Ka Shing Centre for Health Information and Discovery, University of Oxford, Oxford OX3 7LF, United Kingdom; Big Data Institute, Li Ka Shing Centre for Health Information and Discovery, University of Oxford, Oxford OX3 7LF, United Kingdom; Big Data Institute, Li Ka Shing Centre for Health Information and Discovery, University of Oxford, Oxford OX3 7LF, United Kingdom; Big Data Institute, Li Ka Shing Centre for Health Information and Discovery, University of Oxford, Oxford OX3 7LF, United Kingdom

## Abstract

**Summary:**

Ancestral recombination graphs (ARGs) represent the interwoven paths of genetic ancestry of a set of recombining sequences. The ability to capture the evolutionary history of samples makes ARGs valuable in a wide range of applications in population and statistical genetics. ARG-based approaches are increasingly becoming a part of genetic data analysis pipelines due to breakthroughs enabling ARG inference at biobank-scale. However, there is a lack of visualization tools, which are crucial for validating inferences and generating hypotheses. We present tsbrowse, an open-source, web-based Python application for the interactive visualization of the fundamental building blocks of ARGs, i.e. nodes, edges and mutations. We demonstrate the application of tsbrowse to various data sources and scenarios, and highlight its key features of browsability along the genome, user interactivity, and scalability to very large sample sizes.

**Availability and implementation:**

Tsbrowse is installed as a Python package from PyPI (https://pypi.org/project/tsbrowse/), while a development version is maintained at https://github.com/tskit-dev/tsbrowse. Documentation is available at https://tskit.dev/tsbrowse/docs/. Source code is archived on Zenodo with DOI, https://doi.org/10.5281/zenodo.15683039.

## 1 Introduction

Ancestral recombination graphs (ARGs) describe how a set of sample sequences relate to each other at each position along the genome in a recombining species, and are currently the subject of intense research interest (Brandt *et al.* 2024, Lewanski *et al.* 2024, Wong *et al.* 2024, Nielsen *et al.* 2025). ARGs are a fundamental object in population genetics, and although they have been of theoretical interest for decades (Hudson 1983, Griffiths and Marjoram 1996, 1997) it is only with recent breakthroughs in inference methods (Rasmussen *et al.* 2014, Kelleher *et al.* 2019, Speidel *et al.* 2019, Wohns *et al.* 2022, Zhang *et al.* 2023, Deng *et al.* 2024, Gunnarsson *et al.* 2024) that widespread application has become possible. Varied applications have been proposed, such as inferring selection (Stern *et al.* 2019, Hejase *et al.* 2022) and the spatial location of genetic ancestors (Deraje *et al.* 2024, Osmond and Coop 2024, Grundler *et al.* 2025), more powerful approaches to quantifying genetic relatedness (Fan *et al.* 2022, Zhang *et al.* 2023, Gunnarsson *et al.* 2024, Lehmann *et al.* 2025) and other methodological improvements for genome wide association studies (Link *et al.* 2023, Nowbandegani *et al.* 2023), and the development of machine learning methods using inferred ARGs as input (Hejase *et al.* 2022, Pearson and Durbin 2023, Korfmann *et al.* 2024, Whitehouse *et al.* 2024). While these developments are exciting, the performance of these new methods depends critically on the accuracy of the inferred ARGs. Although studies benchmarking the various inference methods on simulated data have emerged (Brandt *et al.* 2022, Deng *et al.* 2024, Peng *et al.* 2025), the practicalities of applying ARG inference to real data are understudied. In particular, there is a critical lack of software infrastructure to support evaluation and quality control of inferred ARGs.

Visualization is fundamentally important to data analysis. Many specialized tools exist to aid the visual analysis and quality control of genome assembly (Wick *et al.* 2015, Challis *et al.* 2020), read mapping (Robinson *et al.* 2011), and variant calling (Robinson *et al.* 2017, Tollefson *et al.* 2019, König *et al.* 2023), e.g. At every stage of a bioinformatics pipeline, it is important to visualize results to avoid artefacts and aid understanding of the data. Genome browsers such as IGV (Robinson *et al.* 2011) and the UCSC Genome Browser (Nassar *et al.* 2023) integrate many different data modalities, and are vital infrastructure for the field.

There is currently no straightforward means of visually summarizing ARGs, presenting a significant stumbling block for the nascent field of practical ARG inference. Inferred ARGs are essentially opaque, with only the most basic numerical summaries (such as numbers of nodes, mutations, etc.) or high-level statistics (Ralph *et al.* 2020) available. While tools for visualizing the local tree topologies exist, they are difficult to interpret and do not scale well to large sample sizes. To address this gap, we present tsbrowse, a client-server application providing genome browser-like functionality for ARGs. It provides interactive visualizations of the information structure of ARGs, smoothly scrolling from chromosome-level views down to individual nodes, edges and mutations. Supporting very large ARGs is a particular focus for tsbrowse, as millions of genome sequences have been sampled for several species (Cesarani *et al.* 2022, Hunt *et al.* 2024, Stark *et al.* 2025) and ARGs of this scale are a particular focus of ongoing research (Kelleher *et al.* 2019, Anderson-Trocmé *et al.* 2023, Zhan *et al.* 2023, Zhang *et al.* 2023, Gunnarsson *et al.* 2024).

## 2 Results

### 2.1 Data model

**Figure 1.**
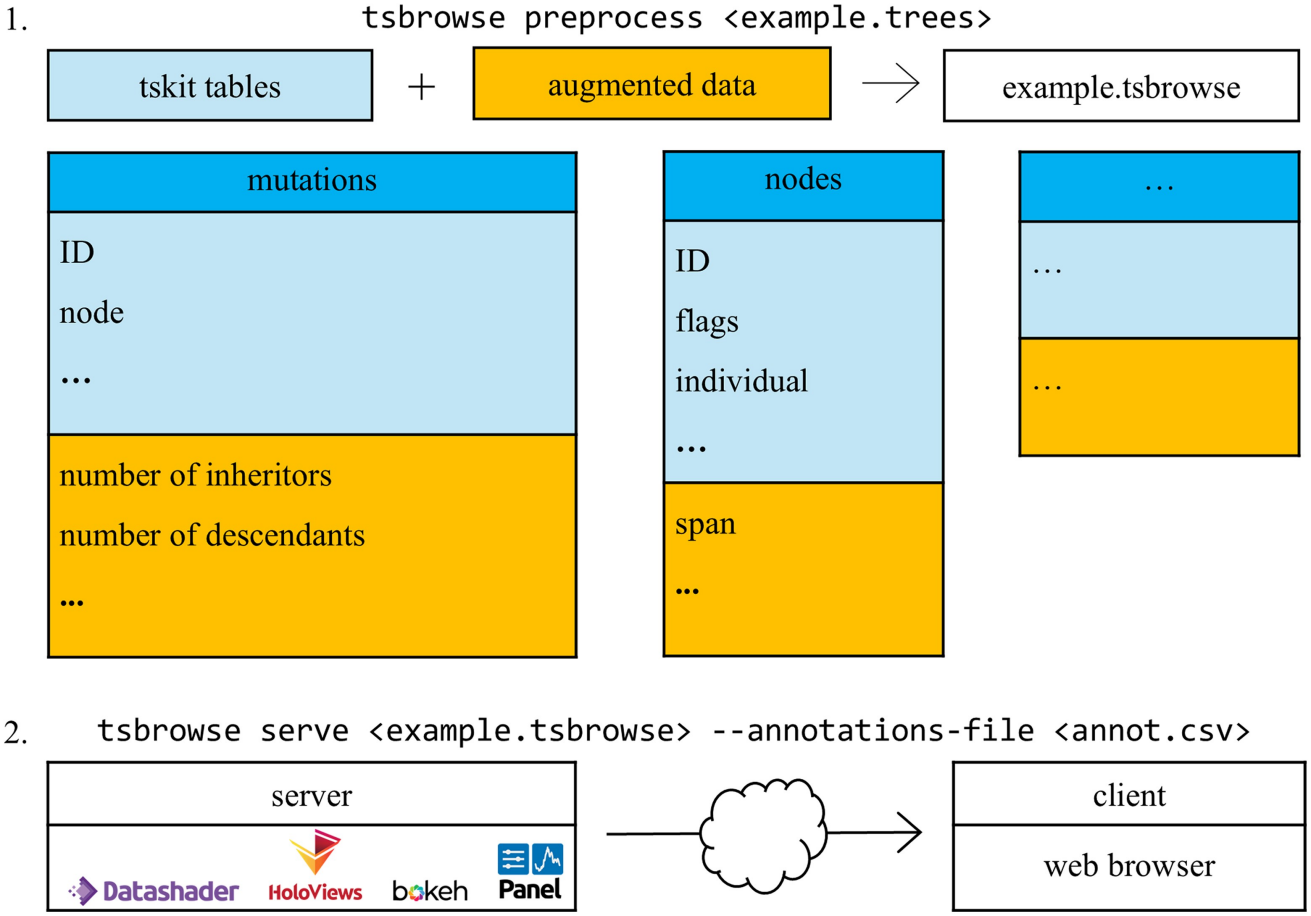
Overview of the tsbrowse architecture. Panel 1 depicts individual tskit tables, with table names in the headers and corresponding ARG properties listed beneath. In the pre-processing step, each table is augmented with additional information computed for visualization, shown as a separate section appended to the table. Panel 2 illustrates the serve step, which renders the output in a web browser using tools from the Holoviz ecosystem. Users may optionally supply an annotation file to overlay contextual information, such as overlapping genes or other sequence features.

Tsbrowse uses the ‘succinct tree sequence’ encoding of ARGs (Wong *et al.* 2024). This efficient ARG encoding is implemented by the tskit library (Ralph *et al.* 2020) and supported by most modern ARG simulation (Kelleher *et al.* 2016, 2018, Haller *et al.* 2019, Adrion *et al.* 2020, Baumdicker *et al.* 2022, Korfmann *et al.* 2023, Lauterbur *et al.* 2023, Tsambos *et al.* 2023, Tagami *et al.* 2024), inference (Kelleher *et al.* 2019, Speidel *et al.* 2019, Mahmoudi *et al.* 2022, Wohns *et al.* 2022, Zhan *et al.* 2023, Zhang *et al.* 2023, Deng *et al.* 2025), and processing methods (Fan *et al.* 2022, Nowbandegani *et al.* 2023). In tskit, ARGs are encoded as a collection of tables, storing information about the nodes and edges that describe the graph topology and the sites and mutations that encode the sequence variation (Fig. 1).

### 2.2 Architecture

Tsbrowse is a modular application written in Python, optimized for ARGs with millions of samples (Fig. 1). The application has two basic commands: preprocess and serve. The preprocess command takes an input ARG file and augments the tskit tables with additional columns, precomputing all the information required for visualization, and storing it as a compressed .tsbrowse file. To visualize an ARG we then run tsbrowse serve, which by default will open a browser window on the local machine, but also supports running the server on a remote machine across the network. This client-server architecture has some important advantages over a monolithic single-machine approach. Most importantly, the ARG being visualized remains in-situ and does not need to be downloaded from the server.

The goal of tsbrowse is to provide interpretable and interactive visual summaries of ARGs containing millions of nodes, edges and mutations. For instance, mutations have a clear interpretation when plotted on genome coordinate versus time axes, but at this scale the data density is far too high to simply plot each mutation as a point. We overcome this problem by using Datashader (Anaconda Developers and Contributors 2024) and the wider Holoviz ecosystem (Holoviz Developers 2024), which efficiently rasterizes large datasets at the requested resolution on the server, sends the image to the web browser for display, and dynamically updates as the user interactively navigates the ARG. This approach allows us to summarize very large ARGs interactively; Fig. 1, available as supplementary data at *Bioinformatics* online e.g. shows a screenshot of tsbrowse summarizing the 1.9 million mutations in a SARS-CoV-2 ARG with around 2.7 million nodes and edges.

### 2.3 User interface

**Figure 2.**
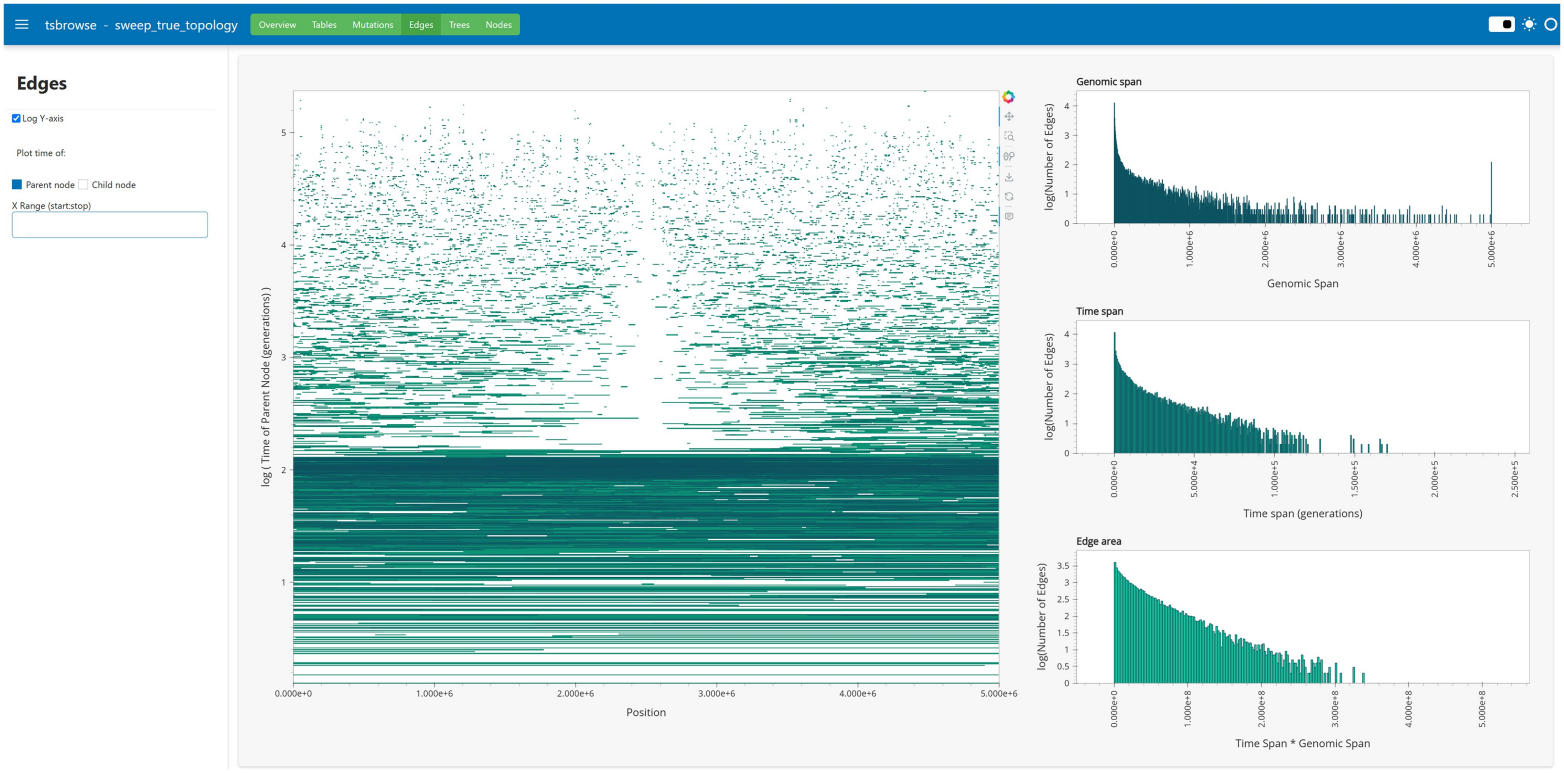
Screenshot of the Edges view in tsbrowse. Visualization of the edges in a simulated ARG with a strong selective sweep in the middle of the genome (see Supplementary, available as supplementary data at *Bioinformatics* online for details). Edges are shown in the main browser pane on the left, with additional histograms summarizing the edges on the right. The effect of the sweep can be seen by the lack of edges crossing the centre of the simulated chromosome due to an excess of recent coalescent events. Moving away from the focal site, the oldest edges are the first to rejoin the ARG, followed by more recent edges, resulting in a wedge-like pattern of missing edges in the centre.

The user interface is presented as a dashboard, with views to describe various aspects of the ARG. Figure 2 demonstrates the user interface using the Edges view as an example. The plot on the left is an overview of the 33 929 edges in a simulated ARG with a strong selective sweep (see Supplement for details). Each edge in the ARG is depicted as a horizontal line connecting the genomic coordinates of the parent and child nodes on the x-axis, and the y-axis shows the time of either the parent or the child, as chosen by the user. The user can interact with the main ‘genome browser’ window using a set of controls on the top-right corner provided by Bokeh (http://www.bokeh.pydata.org), allowing them to pan and zoom as required. The histograms to the right then summarize the edges as depicted in the browser window. A similar browser interface is provided for mutations (e.g. Fig. 1, available as supplementary data at *Bioinformatics* online) and nodes (e.g. Fig. 2, available as supplementary data at *Bioinformatics* online). Tsbrowse also provides an interactive table viewer with flexible searching and sorting utilities, which is a valuable debugging utility for developers.

### 2.4 Applications

The purpose of tsbrowse is to provide an interactive view of the tskit ARG data model to guide intuition, improve inference quality control and facilitate debugging. An example of how we can deepen our understanding using the genome-browser-like perspective provided by tsbrowse is given in the simulated ARG of Fig. 2, where the gap in the Edges view corresponds to the characteristic dip in diversity of a selective sweep. Comparing this ground truth to the ARGs inferred by four different inference methods in Fig. 3, available as supplementary data at *Bioinformatics* online, we can see that there are substantial qualitative differences between the results. These differences in ancestral haplotypes illustrated by the Edges view are unlikely to be captured by the tree-by-tree distance metrics usually used to evaluate inference methods (e.g. Kelleher *et al.* 2019, Zhang *et al.* 2023), providing further motivation for new and improved ways to compare simulated and inferred ARGs (Fritze *et al.* 2024).

Interest in ARG-based methods is burgeoning, but the methods are new, and practical guidance on applying inferences to real data is lacking. Data filtering is essential, and the effects of the choices that must be made along any bioinformatics pipeline on the final ARG are hard to predict and quantify. Tsbrowse was primarily developed as a way to quickly visualize the effects of such filtering choices on ARGs inferred by tsinfer, and it is now an indispensable element of the inference pipeline. Figure 4, available as supplementary data at *Bioinformatics* online shows a region of the 1000 Genomes data with gaps in site density that are spanned by exceptionally long edges, which are likely to bias downstream statistics. These interactive visualizations have also helped diagnose issues with the tsinfer inference algorithm. Figure 2, available as supplementary data at *Bioinformatics* online shows the genomic spans of ancestral nodes in an ARG inferred with tsinfer, demonstrating a clear excess of long haplotypes in the very ancient past. These insights have helped guide development and may lead to significant improvements in performance.

## 3 Discussion

Visualization of tree topology is a central task in phylogenetics, and although numerous tools exist (e.g. Huson *et al.* 2007, Vaughan 2017), the methods can typically only handle a few hundred nodes (but see e.g. Hadfield *et al.* 2018). Visualization of large-scale tree topologies with millions of nodes requires much more sophisticated approaches to capture topological features at different scales, and is an active research area (Wong and Rosindell 2022, Kramer *et al.* 2023). Adapting such methods, and integrating them into tsbrowse to provide a local tree viewer that operates at the million-node scale is an important direction for future work. An interactive viewer for the entire ARG topology, capturing semantic properties of the graph at a range of scales, is an even more ambitious goal, and would be a major asset for the field.

## Supplementary Material

btaf393_Supplementary_Data

## Data Availability

The data underlying this article are available in https://github.com/savitakartik/tsbrowse-paper with DOI: https://doi.org/10.5281/zenodo.16615378
