# Supplementary material for: Tsbrowse: an interactive browser for ancestral recombination graphs
Source: Bioinformatics. 2025 Jul 12;41(8):btaf393. doi: 10.1093/bioinformatics/btaf393 (PMC12342996; doi:10.1093/bioinformatics/btaf393)
Supplement: btaf393_Supplementary_Data [file btaf393_supplementary_data.pdf]

Tsbrowse: an interactive browser for Ancestral Recombination Graphs Supplementary Information

Supplementary Methods

Simulation of truth dataset (Fig. 2)

Ancestral histories of 300 samples were simulated with the `SweepGenicSelection` function in `msprime (version 1.3.3)`. A combination of models was used: in the recent past, a selective sweep was simulated with a beneficial allele situated in the middle of a 5 Mb sequence. The frequency of the allele in the population was set at 0.0001 at the beginning of the sweep. The allele fixed in the population at a frequency of 0.9999. The strength of selection was set using the selection coefficient, *s = 0.25*. A time increment, *dt = 1e-6* was used to step through the sweep. Mutations were added to the ARG at a rate of 1e-8 per base pair per generation. A recombination rate of 1e-8 per base pair per generation was used. For simulating history before the occurrence of the sweep, a standard coalescent model (Hudson's algorithm (Hudson, 1983)) was used until coalescence was achieved.

Inference of SARS-CoV-2 ARGs

The ARG shown in Fig. S1 was inferred with `sc2ts` (Zhan *et al.*, 2023) using the Viridian dataset (Hunt *et al.*, 2024). It consists of 2,482,157 samples, 2,689,054 nodes, 2,689,982 edges and 1,923,169 mutations. Running `tsbrowse preprocess` on the input `tszip` file (113M) required 2m19s of elapsed time (15m15s CPU time) on an Intel Core(TM) i7-9700 CPU. The resulting `.tsbrowse` file size was 130M.

Inference of 1000 Genomes dataset (Fig. S2, Fig. S4)

The 1000 Genomes dataset was downloaded from `https://ftp.1000genomes.ebi.ac.uk/vol1/ftp/data_collections/1000G_2504_high_coverage/working/20220422_3202_phased_SNV_INDEL_SV/`. The ancestral fasta sequence for chromosome 17 (GRCh38) was downloaded from the Ensembl database. Inference was performed with a Snakemake pipeline (`https://github.com/benjeffery/tsinfer-snakemake/`) using `tsinfer version 0.3.3` for the long arm of chromosome 17 after filtering out duplicate variant positions, variants with missing or low quality ancestral allele, singletons, n-1-tons and n-2-tons. Only bi-allelic SNPs were included for inference. For Supplementary Figure 4, `tsdate version 0.2.1` was used to estimate the age of ancestral nodes with *mutation rate = 1.29e-8*, setting all other parameters to default values.

Inference of selective sweep dataset (Fig. S3)

The following software was used to infer ARGs from the truth dataset described in the section "Simulation of truth dataset" above: `tsinfer version 0.3.3` (Kelleher *et al.*, 2019), `tsdate version 0.2.1`, `Relate version 1.2.2` (Speidel *et al.*, 2019), `ARG-needle version 1.0.3` (Zhang *et al.*, 2023), `SINGER version 0.1.8-beta` (Deng *et al.*, 2024). For all inferences, the following parameters were used: *recombination rate = 1e-8*, *mutation rate = 1e-8*, *effective population size = 10,000*. Default values were used for other parameters.

Code availability

Code to recreate datasets used in this paper are available at: `https://github.com/savitakartik/tsbrowse-paper`.

Supplementary Figures

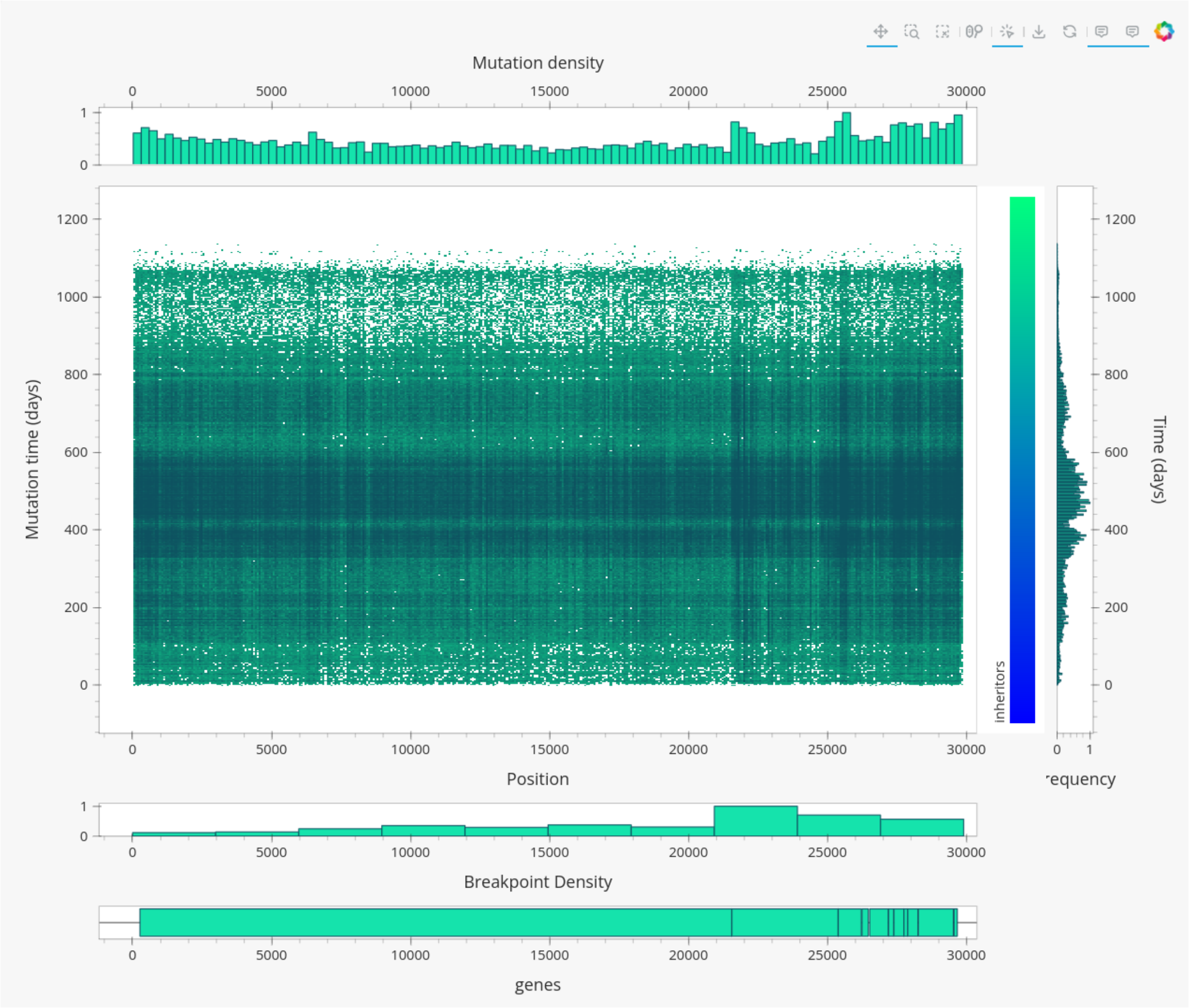

Fig. S 1. `tsbrowse` applied to SARS-CoV-2 ARGs. A screenshot of `tsbrowse`'s depiction of 1,923,169 mutations in an ARG inferred by `sc2ts`; see text for details. Also shown are the gene annotations along the X-axis.

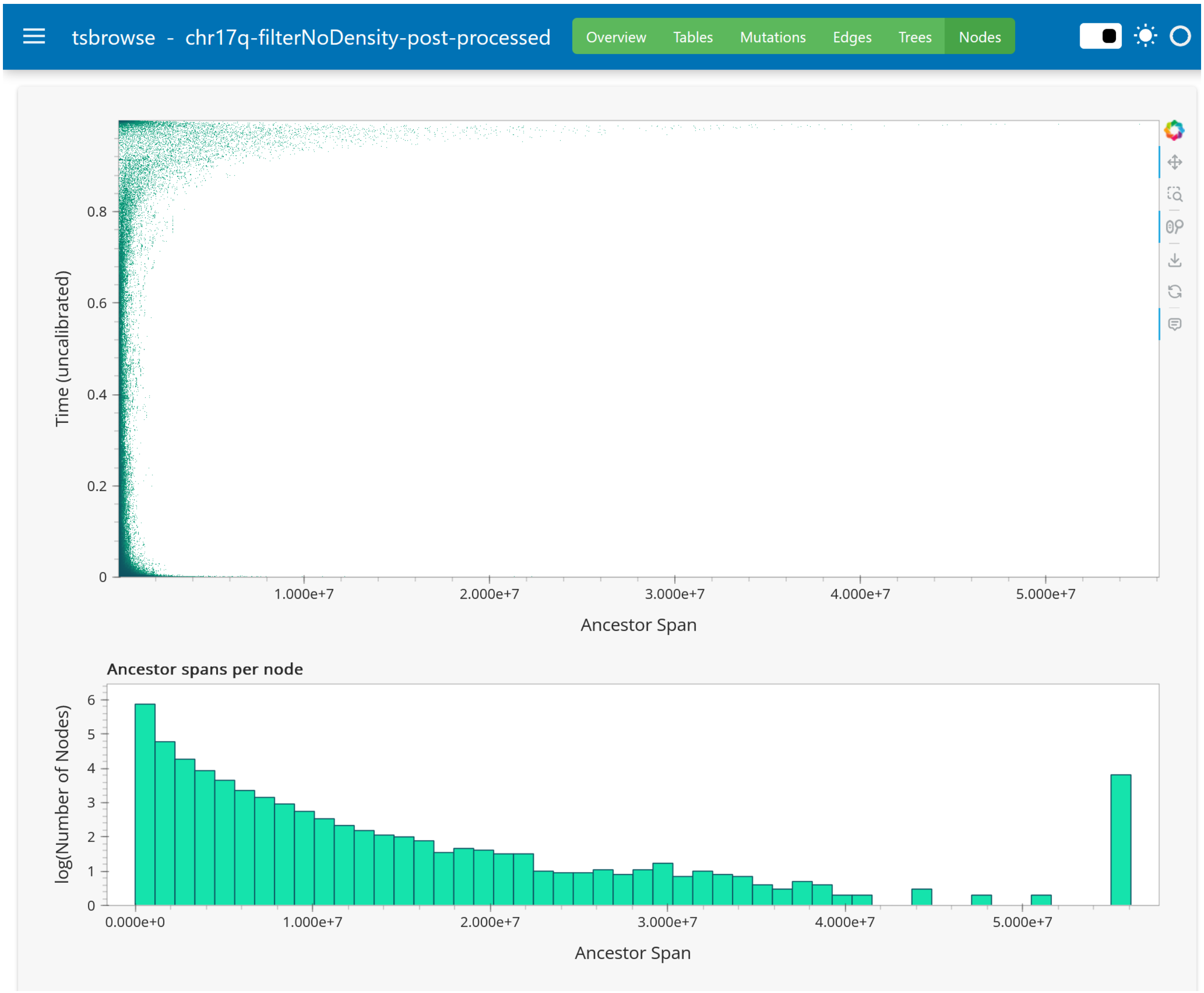

Fig. S 2. Nodes view for a 1000 Genomes inference. A screenshot of `tsbrowse`'s Nodes view for an inference of the long arm of chromosome 17 from the 1000 Genomes whole-genome sequencing dataset. At the top is a plot of node spans over time. The length of sequence that the nodes span is shown on the X axis, and the time of nodes is shown on the Y axis. The histogram at the bottom shows the distribution of node spans.

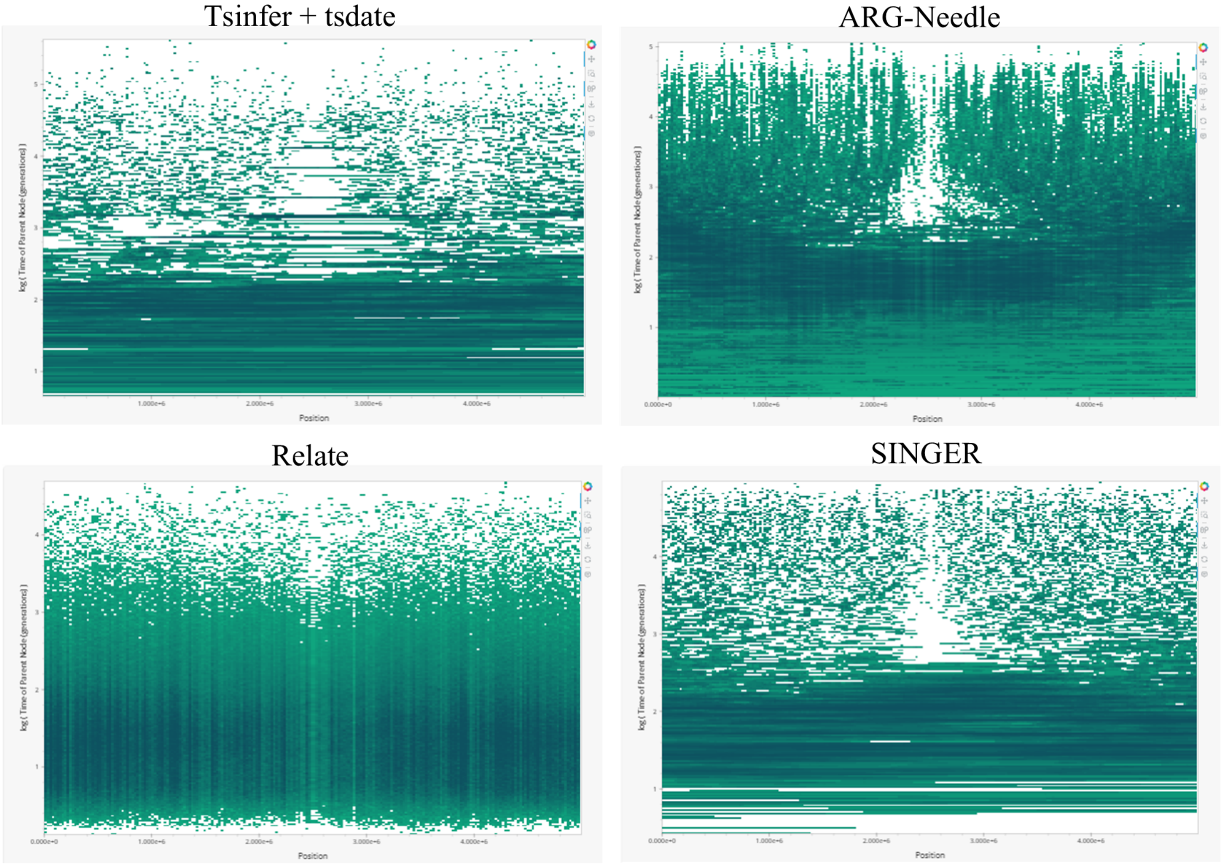

Fig. S 3. `Tsbrowse` applied to inference methods. A screenshot of `tsbrowse`'s Edges view for tsinfer+tsdate, ARG-Needle, Relate and SINGER inferences of the truth dataset simulated under a selective sweep model (shown in Figure 2 of the main text). For SINGER, one of the posterior ARG samples is shown. The X coordinate represents genomic position, each horizontal segment on the plot shows the genomic coordinates that the edge spans, and Y coordinate shows time of either the parent or child node in the edge.

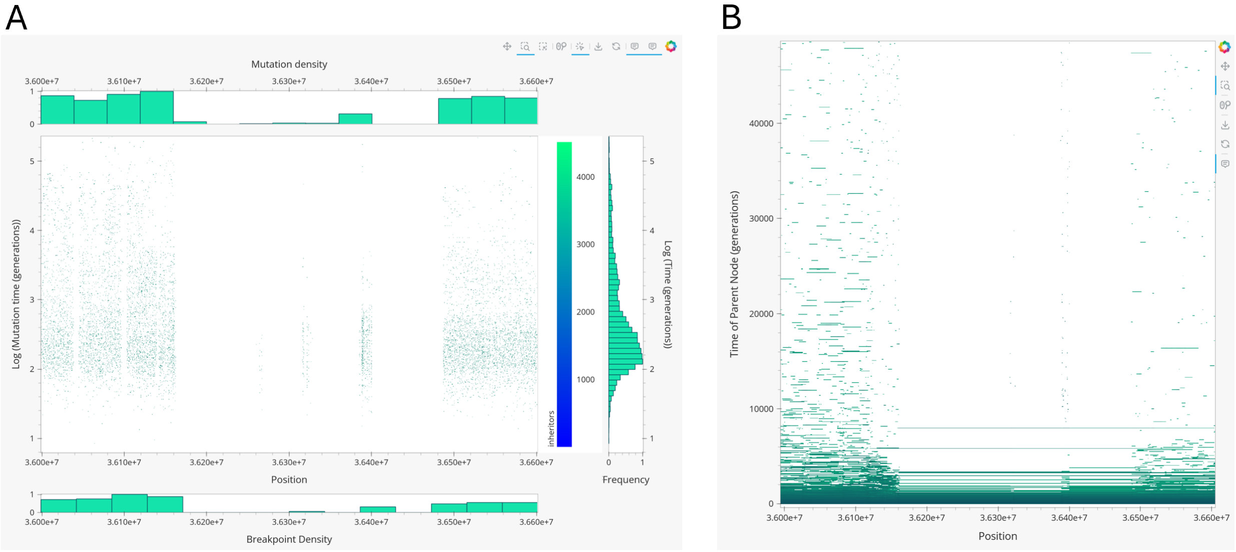

Fig. S 4. Identifying ARG inference problems with `tsbrowse`. Screenshots of `tsbrowse`'s Mutations view (A) and Edges view (B) for a 600 kb region of chromosome 17 inferred from 3,202 participants from the 1000 Genomes Whole Genome Sequencing dataset (Byrska-Bishop *et al.*, 2022). The poor performance of `tsinfer` in this variant-poor region is evidenced by the long edges spanning gaps in mutation density.

References

Byrska-Bishop M., Evani, Uday S., and Zhao X. (2022). High-coverage whole-genome sequencing of the expanded 1000 Genomes Project cohort including 602 trios. *Cell*, **185**.

Deng Y., Nielsen R., and Song Y. S. (2024). Robust and Accurate Bayesian Inference of Genome-Wide Genealogies for Large Samples. *bioRxiv*.

Hudson R. R. (1983). Properties of a neutral allele model with intragenic recombination. *Theoretical Population Biology*, **23**.

Hunt M., Hinrichs A. S., Anderson D., Karim L., Dearlove B. L., Knaggs J., Constantinides B., Fowler P. W., Rodger G., Street T., *et al.* (2024). Addressing pandemic-wide systematic errors in the sars-cov-2 phylogeny. *bioRxiv*.

Kelleher J., Wong Y., Wohns A. W., *et al.* (2019). Inferring whole-genome histories in large population datasets. *Nature Genetics*, **51**(9), 1330–1338.

Speidel L., Forest M., Shi S., *et al.* (2019). A method for genome-wide genealogy estimation for thousands of samples. *Nature Genetics*, **51**, 1321–1329.

Zhan S. H., Ignatieva A., Wong Y., Eaton K., Jeffery B., Palmer D. S., Murall C. L., Otto S. P., and Kelleher J. (2023). Towards pandemic-scale ancestral recombination graphs of sars-cov-2. *bioRxiv*.

Zhang B. C., Biddanda A., Gunnarsson Á. F., *et al.* (2023). Biobank-scale inference of ancestral recombination graphs enables genealogical analysis of complex traits. *Nature Genetics*, **55**.
